# An efficient immunodetection method for histone modifications in plants

**DOI:** 10.1186/1746-4811-9-47

**Published:** 2013-12-16

**Authors:** Geovanny Nic-Can, Sara Hernández-Castellano, Angela Kú-González, Víctor M Loyola-Vargas, Clelia De-la-Peña

**Affiliations:** 1 Unidad de Bioquímica y Biología Molecular de Plantas, Centro de Investigación Científica de Yucatán, Calle 43 No. 130, Col. Chuburná de Hidalgo, Mérida CP 97200, Yucatán, México; 2 Unidad de Biotecnología, Centro de Investigación Científica de Yucatán, Calle 43 No. 130, Col. Chuburná de Hidalgo, Mérida CP 97200, Yucatán, México

**Keywords:** *Agave tequilana*, *Arabidopsis thaliana*, *Capsicum chinense*, *Cedrela odorata*, *Coffea canephora*, Epigenetic mechanism, Histone H3, H3K4me3, H3K9me2, Immunodetection

## Abstract

**Background:**

Epigenetic mechanisms can be highly dynamic, but the cross-talk among them and with the genome is still poorly understood. Many of these mechanisms work at different places in the cell and at different times of organism development. Covalent histone modifications are one of the most complex and studied epigenetic mechanisms involved in cellular reprogramming and development in plants. Therefore, the knowledge of the spatial distribution of histone methylation in different tissues is important to understand their behavior on specific cells.

**Results:**

Based on the importance of epigenetic marks for biology, we present a simplified, inexpensive and efficient protocol for *in situ* immunolocalization on different tissues such as flowers, buds, callus, somatic embryo and meristematic tissue from several plants of agronomical and biological importance. Here, we fully describe all the steps to perform the localization of histone modifications. Using this method, we were able to visualize the distribution of H3K4me3 and H3K9me2 without loss of histological integrity of tissues from several plants, including *Agave tequilana*, *Capsicum chinense*, *Coffea canephora* and *Cedrela odorata*, as well as *Arabidopsis thaliana*.

**Conclusions:**

There are many protocols to study chromatin modifications; however, most of them are expensive, difficult and require sophisticated equipment. Here, we provide an efficient protocol for *in situ* localization of histone methylation that dispenses with the use of expensive and sensitive enzymes. The present method can be used to investigate the cellular distribution and localization of a wide array of proteins, which could help to clarify the biological role that they play at specific times and places in different tissues of various plant species.

## Background

In eukaryotes, including higher plants, the information deposited in the DNA is found compacted into chromatin. However, additional layers of information, known as epigenetics, are imposed on DNA. Furthermore, different molecules can modify the chromatin structure in many ways. For instance, in plants, DNA can be methylated in the deoxycytocine residues in the context of all sequences, from symmetric CG and CHG (where H = A, T or C) to asymmetric CHH [1], whereas histones that conform to the nucleosome (H2A, H2B, H3 and H4) can be covalently modified in their N-terminal tails by methylation, acetylation and phosphorylation, among others [2]. All these modifications are dynamic and reversible and, ultimately, play an essential role in the transcriptional regulation of genes [3,4].

Recent plant genome-wide analysis, particularly in *Arabidopsis thaliana*, rice and maize, has been used to increase knowledge of the plant epigenome and its response to environmental stimuli or developmental cues [5-7]. These studies begin to reveal the correlation between epigenetic marks and transcriptional activity. For instance, it has been shown that the trimethylation of histone H3 at lysine 4 (H3K4me3) is present, most of the time, at the promoter region of genes [5,7,8], while that of the di- and trimethylation of histone H3 at lysine 36 (H3K36me2 and H3K36me3) are distributed over the transcribed region [9,10], and both are abundant in highly expressed genes. By contrast, H3K9me2, a mark related to the formation of heterochromatin, has been found in both the promoter and the gene body [11,12], whereas H3K27me3 is localized within the gene body [13,14]. Both marks (H3K9me2 and H3K27me3) have been associated with the repression of transcription [15].

Several protocols are available to study histones and their modifications, but the majority of them have been done and adapted to the model plant *A. thaliana*[3,16,17]. In addition, other techniques have also been used to examine the spatial distribution of histones and their covalent modifications in the cell, such as fluorescent *in situ* hybridization (FISH) [18] and immunolocalization [19-21]. Although these methods have enabled the visualization of histones’ changes inside the cell, most of the time these methods are realized through complex techniques. These methods may also require sophisticated equipment, such as cryostat to section plant tissue. Another difficulty in these methods is the use of protease inhibitors and several enzymes to degrade the cell wall; these enzymes are typically expensive and sensitive to degradation. Furthermore, the use of squashing during immunolocalization preparation affects the interpretation of the results because the squashing or protoplasting may alter the cellular structures [22,23]. Although these mentioned methodologies have been used in many reports, they do not work well for all plant tissues, as other authors have found.

All these technical issues motivated us to find a simple, suitable and inexpensive protocol to detect *in situ* cellular distribution of histone modifications in a wide array of plants, tissues and conditions (*in vitro* and *ex vitro*). In the present work, we describe step-by-step the required instructions to carry out immunolocalization from paraffin-embedded tissue sections, focusing on several species of agronomical interest such as *Agave tequilana*, *Capsicum chinense*, *Coffea canephora* and *Cedrela odorata*, as well as the model plant *A. thaliana*. These plants were selected to test our protocol because of their economical and agronomical relevance (*A. tequilana*, *C. canephora*, *C. chinense* and *C. odorata*) and their molecular importance as a model (*A. thaliana*). Furthermore, the different behaviors of epigenetic modifications found in these plants could give us a clue about the molecular mechanism that influences their development.

## Results and discussion

### Immunodetection procedure

In order to provide a suitable and standard methodology for *in situ* immunolocalization from FAA-fixed and paraffin-embedded plant tissues, we developed a simple and reproducible procedure for *in situ* immunolocalization in several tissues and plants, improving some of the steps reported by other protocols [23-27]. For instance, the use of only one step during the protein-antibody interaction has increased the antibodies’ efficiency in several tissues in different plant growth conditions. Also, the time of fixation in different kinds of tissues was homogenous in all samples, even for those plants with rigid cell walls and particularly for timber species such as coffee and cedar. In addition, we have discarded the use of enzymes responsible for degrading the cell wall, and the use of dimethylsulfoxide, NP40 and other reagents frequently used to achieve the cellular permeabilization [22,23,28], which increase the cost of the immunolocalization protocols. Instead of all the previously listed chemicals, we used a single step for the recovery of antigen sites without affecting the histological integrity, obtaining a clear-cut protein distribution.

**Figure 1:**
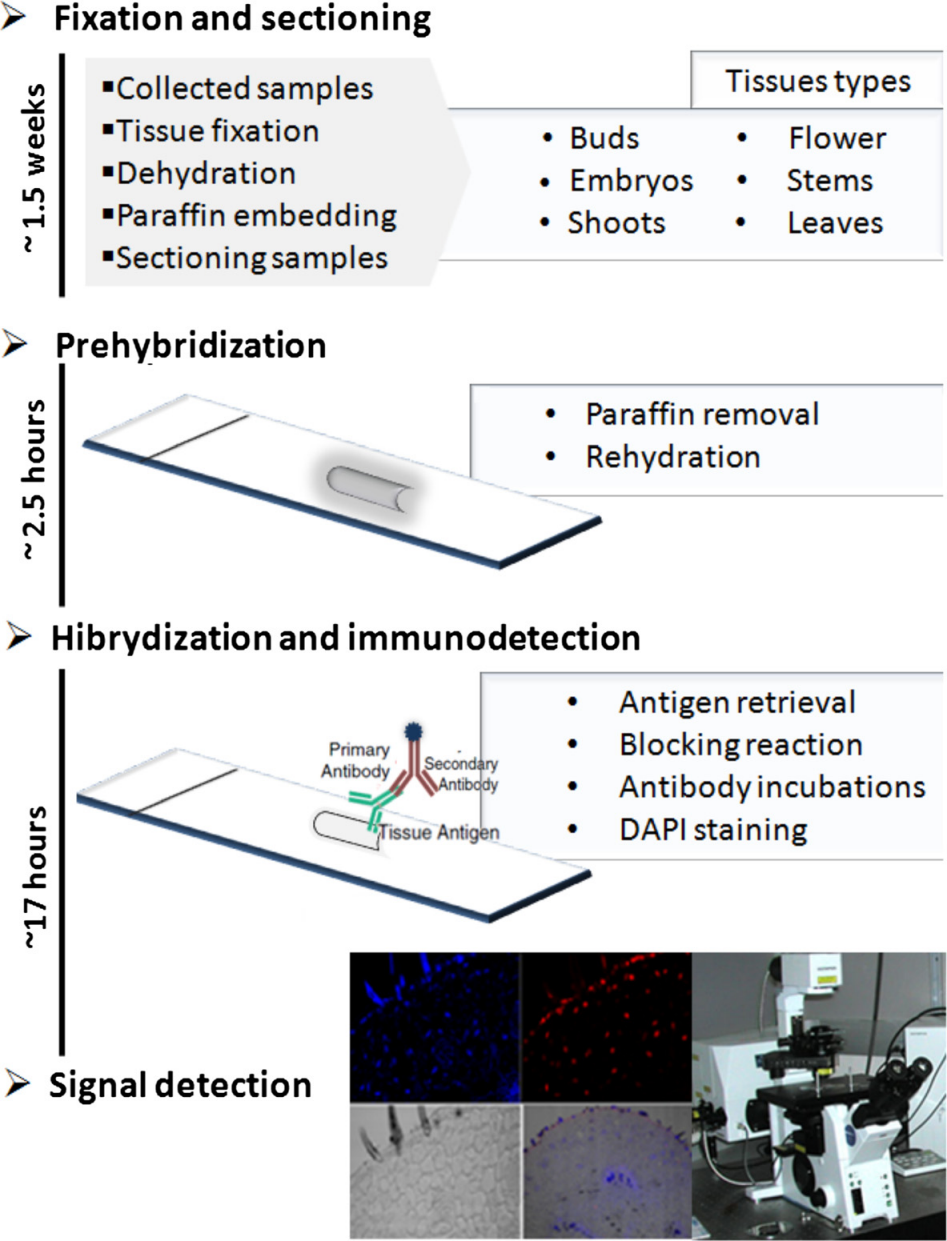
**Schematic representation of the principal steps performed in the protocol for immunodetection of histone methylation.** Different tissues were collected and fixed with FAA solution and paraffin-embedded. The samples were sectioned into 4-5-μm slices and the slides were dewaxed. The antigenic sites were retrieved and the tissue was blocked and incubated with the primary and secondary antibodies. The samples were counterstained with DAPI and analyzed under confocal microscope.

**Figure 2:**
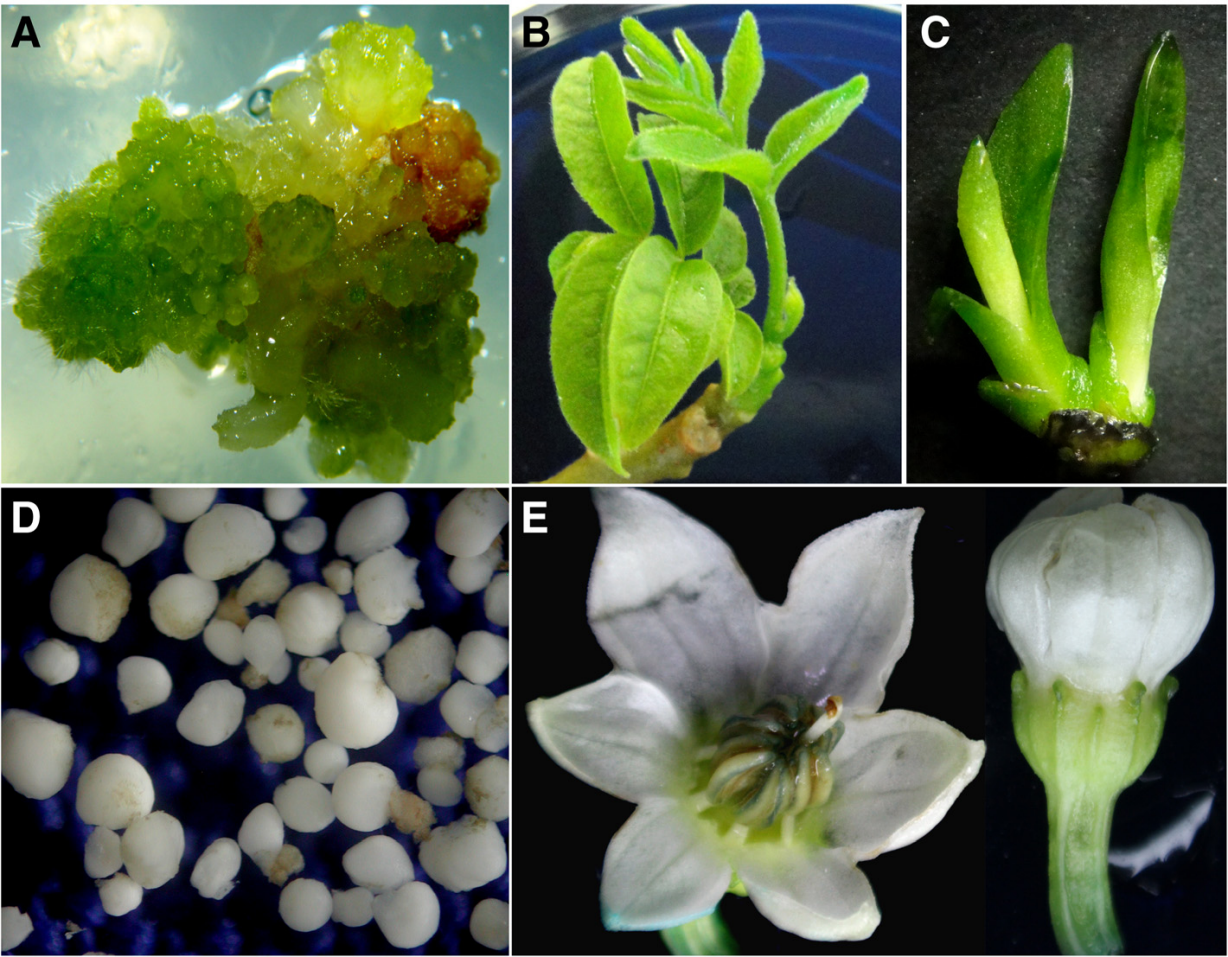
**Plant material collected for the immunodetection proteins.(A)** Callus of *Arabidopsis thaliana*. **(B)** Shoots and buds of *Cedrela odorata*. **(C)** Plantlets of *Agave tequilana*. **(D)** Somatic embryos of *Coffea canephora* obtained 56 days after induction. **(E)** Flowers of *Capsicum chinense*. **A**, **C** and **D** were obtained through cultures *in vitro*. **B** and **E** were collected from the field.

To test the reproducibility of our method, several tissues of different plant species of economical and agronomical interest, such as Agave, chili pepper, coffee, cedar and Arabidopsis were collected and treated under the procedures summarized in Figure 1. Tissues of several plant species from both *ex vitro* and *in vitro* conditions (Figure 2) were fixed in formaldehyde. In most of the protocols, the fixation step must be optimized according to the type of plant or tissue [23,28]. In the protocol described here, the sample fixation was carried out with final concentration of 3.7% formaldehyde, providing good results. In addition, the formaldehyde promotes a strong preservation of the cellular and chromosomal structure. After the fixation step, the samples were dehydrated and paraffin-embedded in order to obtain a solid sample that would maintain tissue integrity during the sectioning step. Once the sliced sections are obtained, the tissue can be used for subsequent probes or stored at 4°C for several months without loss of integrity.

In order to remove the paraffin from the tissue, the tissue sections were heated at 65°C and rinsed with xylene and then with ultraclear. These steps are needed to allow full elimination of the paraffin residues; otherwise, this substance presents one of principal interferences in the immunodetection process. After total paraffin removal, the tissue samples were rehydrated and the antigen retrieval was performed. This step represents a critical point, because it enables the antibody to access the protein antigens, thus increasing signal intensity during immunodetection [29].

**Figure 3:**
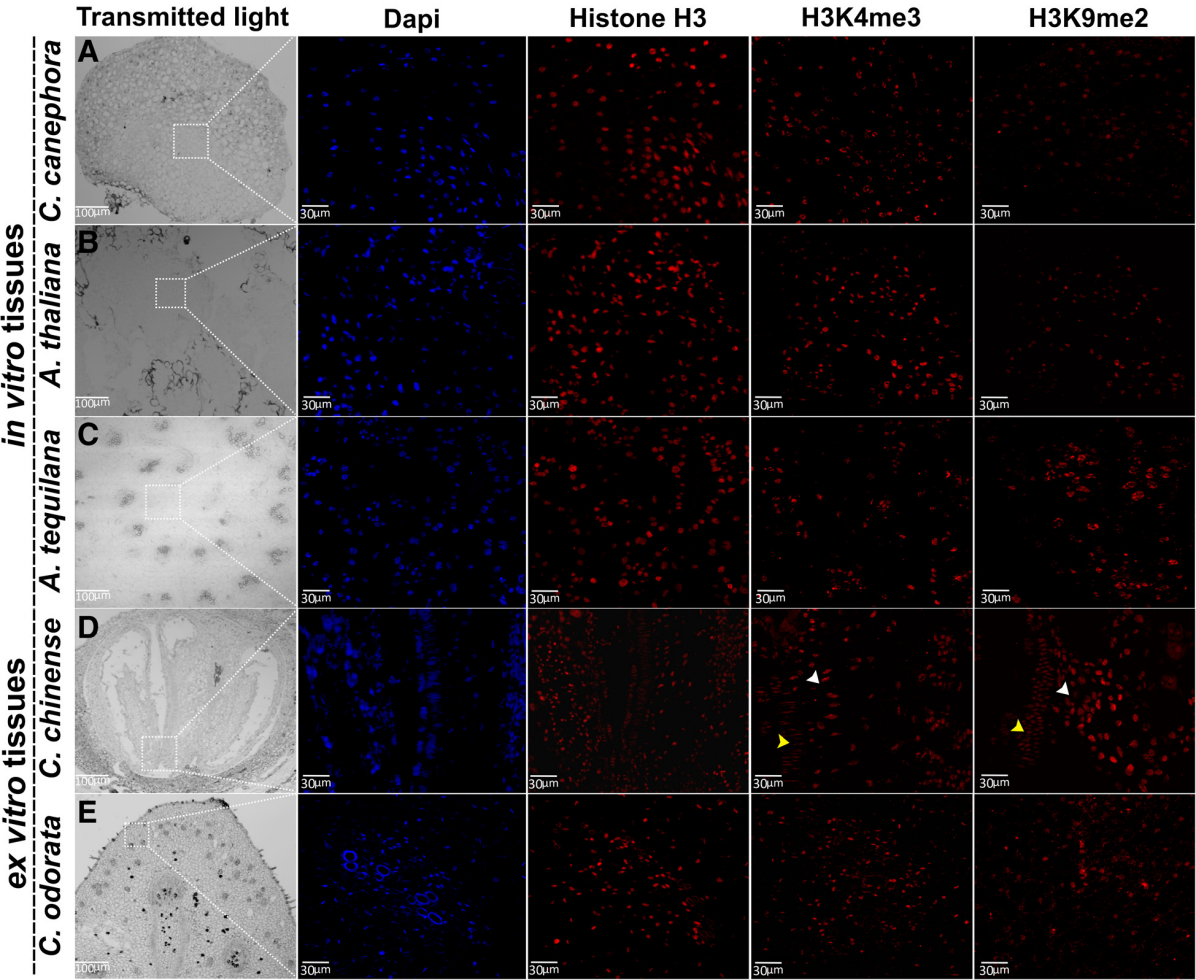
**Immunodetection of histone modifications in tissues of several plant species.** Immunodetection was performed using antibodies against H3K4me3 and H3K9me2, using the histone H3 as positive control. Panels from left to right show the confocal images of transmitted light, DAPI, histone H3, H3K4me3 and H3K9me2. Dashed squares represent the close-up of the sites detected by immunoflourescence with the antibodies mentioned above in the globular embryo of *Coffea canephora***(A)**, in meristematic zones in the callus of *Arabidopsis thaliana***(B)**, in the shoot apex of *Agave tequilana***(C)**, in the endothecium (yellow arrowhead) and in the lobes cells of anthers (white arrowhead) in *Capsicum chinense***(D)** and in the proximal cells to epidermis of *Cedrela odorata ***(E)**.

By nature, some protein epitopes are hidden within the complex structure of proteins, making it difficult to visualize the results during the immunolocalization. In several protocols of *in situ* hybridization or immunodetection in plants, the use of proteases or enzymes to degrade the cell wall is necessary in order to increase the permeability of the cell and its contents [23,28,30]. However, these techniques sometimes fail to achieve an appropriate immunostaining for many proteins. A suitable alternative for the development of antigen retrieval without the use of enzymes, which has been applied for clinical proposes in humans [29], is through boiling paraffin-embedded sections in the proper buffer. This procedure has the advantage of unmasking the tissue antigens, improving signal detection. Therefore, we investigated whether this procedure can be used in plants. We found that the heat-induced antigen retrieval using microwaved citrate buffer can indeed be applied to plant tissue without loss of histological integrity or protein detection. This step did not affect the tissue integrity in any of the plant species used here (Figure 3; transmitted light) and this step seems to be essential prior to the detection phase. Several protocols indicate that microwave irradiation enhances antigen retrieval, and it has been shown that this procedure is superior to traditional immunoflourescence in mammals [31,32]. Microwave irradiation has been used in plant tissues [33-35], and the use of this procedure improves the fixation process at low concentrations of formaldehyde. It is known that during heating, the energy provided helps to break some of the bonds formed during the fixation, increasing the number of available antigens in the cells and thus improving signal intensity [24,36]. On the other hand, some reports have proposed that the use of methanol/acetone solution can preserve cellular architecture, allowing better access of the antibody to the antigen [22,23]. We tested this option; however, we did not find satisfactory results in most of the plants used (data not shown). Therefore, the antigen retrieval from the microwaved citrate buffer was chosen as a critical and essential step for immunodetection in all species studied here. We have found that the use of citrate solution with the microwave heating treatment increases the immunoreactivity, since the tissues without microwave-citrate treatment showed a substantial decrease or absence of signal (Additional files 1, 2 and 3).

Another key factor that directly impacts the immunodetection is the quality of the primary antibodies, because good results obtained by Western blot do not always provide positive results for immunodetection. Therefore, selected monoclonal or polyclonal primary antibody dilutions need to be tested. We obtained satisfactory results using both monoclonal and polyclonal antibodies at a 1:200 dilution, a process which has been tested previously by Western blot and chromatin immunoprecipitation with different plant species [37,38]. On the other hand, the fluorescence-labeled secondary antibody is also important for the final signal detection, but this type of antibody needs to be chosen according to the microscope filter set and the working dilution should be determined experimentally. Here, Alexa Fluor® 647 at a 1:100 dilution provided good results. It must be noted that once the tissue sections are incubated with the fluorescence-labeled secondary antibody, they should be protected from light during all washes, as well as during DAPI staining and before to confocal analysis. In all steps, it is also important to prevent the tissue from drying, in order to decrease background, false signals or artifacts.

### Versatility of the immunodetection method in several plants, tissues and conditions

According to the efficient and reproducible method described above, the results obtained by this protocol show that different plant species (Agave, chili pepper, cedar, coffee and Arabidopsis) can be treated with the same methodological conditions without loss of histological integrity, allowing a good recovery of antigenic sites and culminating with a better signal resolution. We collected material of both *ex vitro* and *in vitro* plant tissues, such as *A. thaliana* callus (*in vitro*) (Figure 2A), cedar buds (*ex vitro*) (Figure 2B), Agave plantlet stems (*in vitro*) (Figure 2C), coffee somatic embryos (*in vitro*) (Figure 2D) and chili pepper flowers (*ex vitro*) (Figure 2E), and, in all cases, the reproducibility of our protocol for histone methylation localization was noted.

It was also observed that the signal intensity in all tissues of histone H3 and DAPI was well defined. Furthermore, a clean and clear signal of histone H3 between the cell localization of histone H3 and its localization inside the nucleus was observed (Figures 3). To discard the effect of autofluorescence, tissue sections were exposed without primary antibodies, which served as the negative control for both tissue sections as well as secondary antibody nonspecificity (data not shown). One of the most studied histone marks in plants and animals is H3K4me3, which is related to gene expression and, therefore, very dynamic [7,8]. In contrast, H3K9me2 represents a gene-silencing mark that promotes heterochromatin formation [39].

**Figure 4:**
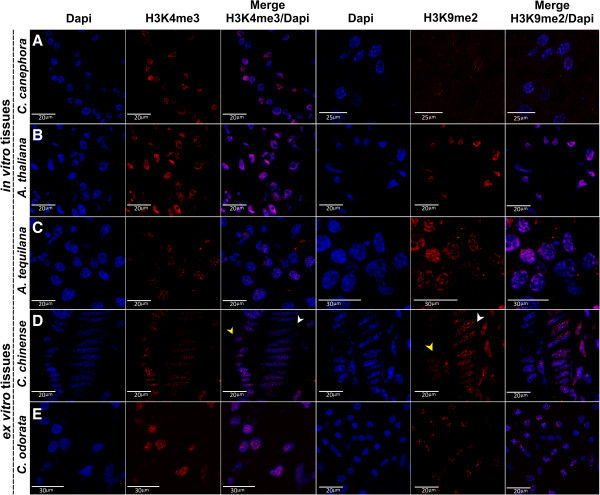
**Visualization of H3K4me3 and H3K9me2 in specific cells in different plants.** Immunodetection was carried out with specific antibodies against H3K4me3 and H3K9me2. Confocal images of DAPI, H3K4me3, H3K9me2 and merged signals are shown from left to right and represent specific zones magnified from dashed squares shown in Figure 3, to verify that the signal in section tissues is free of background noise and it is inside cells. **(A)** Meristematic cells of globular embryo in *Coffea canephora*. **(B)** Callus cells of *Arabidopsis thaliana*. **(C)** Shoot apex cells of *Agave tequilana*. **(D)** Epidermic layer (yellow arrowhead) and endothecium cells (white arrowhead) of lobes anthers in *Capsicum chinense*. **(E)** Epidermis and collenchyme cells of *Cedrela odorata*. In all species, the H3K4me3 was observed to be particularly abundant in the biggest and largest cells; whereas the H3K9me2 was visualized in the nuclei of cells preferentially as flourescent spots.

To test our protocol, we observed the distribution of antagonistic marks (H3K4me3 and H3K9me2) in several plant tissues and conditions (Figures 3 and 4). It was found that both epigenetic marks were highly dynamic, and these presented different subcellular distributions depending on the tissue of each plant species. For instance, in the somatic embryo at the globular stage of *C. canephora*, the results revealed that spatial distribution of H3K4me3 was significantly different in comparison with H3K9me2, which presented a slight decrease in its signal in the center of this tissue, the region where the procambium cells would establish (Figure 3A). In the callus of *A. thaliana*, it was observed that the presence of H3K4me3 was prominent in those cells located at the meristematic zones (Figure 3B), while the H3K9me2 had lower signal. In the case of the transversal cut of *A. tequilana* plants, the highest levels of H3K9me2 were detected in cells of the shoot apex in comparison to H3K4me3 (Figure 3C).

In the *C. chinense* flower, a longitudinal cut revealed a low signal of H3K4me3 in the endothecium cells, as well as in the cells of the central lobes of the anthers. However, the levels of H3K9me2 in the same cells were higher than H3K4me3 (Figure 3D). In the vegetative bud of *C. odorata*, specifically in the collenchyma cells and epidermis, the levels of the H3K4me3 and H3K9me2 were similar (Figure 3E).

The cellular details observed with this method show, in a more detailed resolution (Figure 4), that the signal of H3K4me3 and H3K9me2 inside the nucleus of the cells was free of background noise and very specific. For instance, it was found that the cells that contain the strong signal intensity of H3K4me3, which is related to transcriptional activity, was localized mainly in big nucleus, while that of H3K9me2 was observed in the compacted nucleus, particularly restricted to heterochromatic zones (Figure 4).

In the case of the globular embryo of *C. canephora*, the signals of H3K4me3 in the inner cells were clearly visible, which was consistent with the low signal of H3K9me2; this probably suggests a transcriptional activity in this area (Figure 4A). This same pattern was also observed in the *A. thaliana* callus (Figure 4B) in the central zone of the meristematic region. On the other hand, in the meristematic cells of the *A. tequilana* shoot apex, a low signal of H3K4me3 was detected, while high signals of H3K9me2 were seen (Figure 4C).

In *C. chinense*, high levels of H3K9me2 were observed in the endothecium cells (subepidermal layer that develops fibrous wall thickenings in late anther development) but only a slight increase of H3K4me3 in the epidermis cells of the anther lobes was detected. These results could be related to the immature anther, because even the epidermal layer is transcriptionally active, but that frequently disappears before anther maturity to expose the endothecium [40]. In the case of *C. odorata*, it was possible to detect the distribution of high levels of H3K4me3 in the epidermis cells, whereas H3K9me2 occurs preferentially in the collenchyma cells of the bud (Figure 4E). These results provide information about the cell types and their possible activity during the vegetative growth of the buds.

The results presented in this work propose an efficient immunolocalization method that can be used in different plant tissues and plant growth conditions. Moreover, deposition of the epigenetic marks H3K4me3 and H3K9me2 were found in different tissues, including callus, flowers, buds, somatic embryos and vascular procambium, as well as in different conditions (*in vitro* and *ex vitro*). The differential localization of the epigenetic marks analyzed with this protocol revealed the complex spatial distribution of H3K4me3 and H3K9me2 that could indicate the efficient use of epigenetic mechanisms by different types of plants and tissues to regulate gene transcription during their growth and development or in response to environmental stimuli [7,27].

## Conclusions

Here we provide an easy, efficient and inexpensive method to carry out the immunodetection of histone methylation; it is likely to be suitable also for use with a wide spectrum of antibodies. This method has been shown to be effective with several plants of agronomical and biological importance, such as coffee, agave, chili pepper, cedar and *Arabidopsis*, and in controlled environmental conditions (*in vitro*), or in looking at plants exposed to uncontrolled conditions or that are difficult to cultivate in the laboratory, such as cedar (*ex vitro*). Furthermore, in this work, we have shown that antigen retrieval performed by microwave heating and the use of citrate buffer improves the immunodetection process and enhances signal detection and the quality of the immunolocalization. The spatial distribution and cellular localization of the proteins of interest can be obtained by this simple, highly reproducible and inexpensive method, which could be applied to different tissues of other plant species.

## Methods

### Plant material

*Cedrela odorata* plantlets and flowers of *Capsicum chinense* were collected at the greenhouse at CICY, Mérida, Yucatán, México. *In vitro Agave tequilana* plantlets were cultivated in Murashige & Skoog [41] medium supplemented with 10 mg/L BAP and 0.025 mg/L 2,4-D and cultured under photoperiod conditions (16 hours of light/8 hours of dark at 25°C). Callus of *Arabidopsis thaliana* (ecotype Col-0) were established from immature zygotic embryos according to Mordhorst et al. [42] and cultured under the same photoperiod conditions described above. Somatic embryos of *Coffea canephora* were obtained according to the method described by Quiroz-Figueroa et al. [43].

### Reagents

Albumin from bovine serum (BSA) was obtained from Sigma (Cat. # A2153). Anti-histone H3 antibody, CT, pan (α-H3; Cat. # 07–690), anti-trimethyl-histone H3 at lysine 4 (α-H3K4me3; Cat. # 05-745R) and anti-dimethyl-histone H3 at lysine 9 (α-H3K9me2; Cat. # 07–441) were purchased from Millipore. Alexa Fluor® 647 goat anti-rabbit IgG was obtained from Invitrogen (Cat. # A21244). VECTASHIELD® mounting medium with 4,-6-diamidino-2-phenylindole (DAPI) was obtained from Vector laboratories (Cat. # H-1200).

### Immunodetection protocol

Each of the plant tissues was collected and fixed in FAA solution [10% formaldehyde (Fischer BioReagents®, BP531), 5% acetic acid (Sigma, 695092), 50% absolute ethanol (Meyer®, 0390)] for 48–72 h at 4°C, changing the FAA solution every 24 h and applying vacuum for 10 min at each change. Then, the FAA was removed and the samples were dehydrated through a gradual increase of 30, 50, 70, 85% (2 × 2 h each) and 96% (2 × 30 min each) of absolute ethanol, applying vacuum for 5 min at each step and maintaining the samples at 4°C. After that, the samples were incubated twice for 24 h each in 100% butanol (J.T. Baker, 9054–03) at room temperature.

To begin the process of embedding the tissue samples in paraffin, fresh butanol and 15–30 flakes of Paraplast Plus® (Sigma, P3683) were added to the samples and incubated overnight at room temperature at 60 rpm. Then, the samples were incubated at 60°C, adding 30–40 flakes of paraffin every 2–3 hours, three times. The excess butanol was removed changing the liquid paraffin every 12 hours, four times. Tissue samples were placed in the center of stainless steel base molds (Fisher Scientific, 15-182-505B) previously heated to 60°C and embedded in paraffin to fill; then, the molds were covered with the cassettes’ hold tissue (Fisher Scientific, 15182702A). The cassettes were removed after 3–4 h at room temperature and the paraffin-embedded sample was sectioned into 4–5 μm slices using a retracting microtome (MICROM®, HM 325) with low profile blades (Thermo Scientific, 1407060). The sections were placed in a 42°C water bath to allow the correct expansion of the tissues, and then placed on micro slides (Sigma, S8400). Tissue sections attached to the micro slides were maintained at 37°C at least for 2 hours. From this step, the tissue sections can be stored at 4°C for several months without losing tissue integrity.

Paraffin removal was accomplished as follows: dried sections were incubated at 65°C for 15 min and deparaffinized in slide staining jars (Fisher Scientific, 08-813E) with xylene, (Sigma, 534056) three times for 10 min per rinse, and ultraclear (J.T. Baker, 3905), four times every 15 min per rinse. The ultraclear was removed and the slides were washed twice in absolute ethanol at 100% for 2 min per rinse. Then, the tissue sections were rehydrated with a series of absolute ethanol-water combinations (96, 80, 70, 50 and 30% for 5 min in each step) and water twice (5 min each).

Antigen retrieval was carried out by rinsing the slides in citrate buffer [10 mM Sodium citrate dehydrate (Sigma, W302600) at pH 6 adjusted with 1 N of HCl, and 0.05% Tween® 20 (Sigma, P1379); this solution can be stored at 4°C] and microwave-heated at high power for 4 min. In order to prevent tissue drying during the microwave exposure, the amount of buffer needs to be enough to cover the sample. After heating, the slides were rinsed with warm water to slowly cool the temperature of the slide over 5 min and, then, the slides with the sample tissue were washed three times with PBS buffer [150 mM sodium chloride crystal (J.T. Baker, 3624–01), 10 mM sodium phosphate dibasic (Sigma, S3264), 2 mM potassium phosphate monobasic (Sigma, P5655) pH 7.2 adjusted with 1 N NaOH] for 5 min each. The PBS buffer should be freshly prepared and filtered through a 0.45-μm membrane.

Excess of PBS was carefully removed around the tissue section in the slides with absorbent paper and incubated with 20–30 μl of blocking buffer (3% BSA diluted in PBS buffer) avoiding bubbles. The slides were incubated in a wet chamber for 1 hour at 4°C [wet chamber can be prepared with a moistened Whatman paper placed on the bottom of a petri dish (Sigma, CLS3160100) and tightly sealed with paraffin film]; then, the slides were washed three times with PBS buffer for 5 min each. The excess of PBS was removed again and 20–30 μl of the primary antibody (anti-H3, anti-H3K4me3 and anti-H3K9me2) was added to each tissue section, and then the slides were incubated overnight at 4°C in a wet chamber. After the washes in PBS buffer (3 × 5 min), the PBS was removed and the sections were incubated with 20–30 μl of the fluorescently labeled secondary antibody Alexa Fluor® 647 for another 3 hours in a wet chamber at room temperature. The dilution of both primary and secondary antibodies was performed in 1% BSA diluted in PBS buffer at 1:200 and 1:100, respectively. Subsequent washes in PBS buffer (3 × 10 min) were carried out while protecting the slides from the light. Finally, the tissue sections in the slides were counterstained with 20 μl of Vectashield® mounting medium, with DAPI to stain the DNA, mounted with a 22 × 22 mm cover glass (Sigma, C9802) and sealed around the perimeter with nail polish or a plastic sealant and stored in the dark at 4°C prior to analysis. The photographs were obtained using a confocal laser scanning microscope (Olympus, FV100 SW) and the FV10 ASW 3.1 viewer software. The H3, H3K4me3 and H3K9me2 signals were detected using an excitation wavelength at 650 nm, and the DAPI staining signal was detected using the excitation wavelength of 405 nm.

## Abbreviations

H3: Histone H3; H3K4me3: Trimethyl-histone H3 at Lys4; H3K9me2: Dimethyl-histone H3 at Lys9; BSA: Albumin from bovine serum; DAPI: 4,-6-diamidino-2-phenylindole.

## Competing interests

The authors declare that they have competing interests.

## Authors’ contributions

GNC conceived the protocol, collected the tissue samples, did the immunodetection and drafted the manuscript. SHC performed the histology of samples and helped with immunodetection assays. AKG performed the confocal image analysis. VMLV and CDP coordinated the project, helped with the design of the study and assisted with the drafting of the manuscript. All authors read and approved the final manuscript.

## Supplementary Material

Additional file 1 **Immunodetection of histone H3 in different plant species tissues without the microwave treatment (negative control).** Immunodetection against histone H3 avoiding the antigen retrieval from the microwaved citrate buffer in the globular embryo of *Coffea canephora* (A), meristematic zones in the callus of *Arabidopsis thaliana* (B), procambium zone of *Agave tequilana* (C), anthers of *Capsicum chinense* (D) and bud of *Cedrela odorata* (E). Click here for file

Additional file 2 **Immunodetection of H3K4me3 in different plant species tissues without the microwave treatment (negative control).** Immunodetection against H3K4me3 avoiding the antigen retrieval from the microwaved citrate buffer in the epidermis cells of globular embryo of *Coffea canephora* (A), meristematic zones in the callus of *Arabidopsis thaliana* (B), shoot apex of *Agave tequilana* (C), anthers of *Capsicum chinense* (D) and in the proximal cells to epidermis of *Cedrela odorata* (E). Dashed squares represent the close-up of the sites analyzed by immunodetection against H3K4me3 without antigen retrieval. Click here for file

Additional file 3 **Immunodetection of H3K9me2 in different plant species tissues without the microwave treatment (negative control).** Immunodetection against H3K9me2 avoiding the antigen retrieval from the microwaved citrate buffer in the epidermis of globular embryo of *Coffea canephora* (A), meristematic zones in the callus of *Arabidopsis thaliana* (B), shoot apex of *Agave tequilana* (C), anthers of *Capsicum chinense* (D) and in the proximal cells to epidermis of *Cedrela odorata* (E). Dashed squares represent the close-up of the sites analyzed. Click here for file

## References

[B1] Vanyushin B Ashapkin VV DNA methylation in higher plants: past, present and future Biochim Biophys Acta 1809 9 360 368 10.1016/j.bbagrm.2011.04.006 21549230

[B2] Kouzarides T Chromatin modifications and their function Cell 2007 9 693 705 10.1016/j.cell.2007.02.005 17320507

[B3] Zhang K Sridhar V Zhu J Kapoor A Zhu JK Distinctive core of histone post-translational modification patterns in *Arabidopsis thaliana* Plos One 2007 9 e1210 10.1371/journal.pone.0001210 18030344 PMC2075165

[B4] Bannister J Kouzarides T Regulation of chromatin by histone modifications Cell Res 2011 9 381 395 10.1038/cr.2011.22 21321607 PMC3193420

[B5] Li X Wang X He K Ma Y Su N Stolc V High-resolution mapping of epigenetic modifications of the rice genome uncovers interplay between DNA methylation, and gene expression Plant Cell 2008 9 259 276 10.1105/tpc.107.056879 18263775 PMC2276441

[B6] Li H Freeling M Lisch D Epigenetic reprogramming during vegetative phase change in maize Proc Natl Acad Sci USA 2010 9 22184 22189 10.1073/pnas.1016884108 21135217 PMC3009802

[B7] Zhang X Bernatavichute YV Cokus S Pellegrini M Jacobsen S Genome-wide analysis of mono-, di- or trimethylation of histone H3 lysine 4 in Arabidopsis thaliana Gen Biol 2009 9 R62 10.1186/gb-2009-10-6-r62 PMC2718496 19508735

[B8] Stimpson K Sullivan B Histone H3K4 methylation keeps centromeres open for business EMBO J 2011 9 233 234 10.1038/emboj.2010.339 21245889 PMC3025472

[B9] Xu L Zhao Z Dong A Taconnat L Renou J Steinmetz A Di- and tri- but not monomethylation on histone H3 lysine 36 marks active transcription of genes involved in flowering time regulation and other processes in *Arabidopsis thaliana* Mol Cel Biol 2008 9 1348 1360 10.1128/MCB.01607-07 PMC2258740 18070919

[B10] Sui P Jin J Mu C Gao J Feng H Shen W H3K36 methylation is critical for brassinosteroid-regulated plant growth and development in rice Plant J 2012 9 340 347 10.1111/j.1365-313X.2011.04873.x 22136623

[B11] Naumann K Fischer A Hofmann I Krauss V Phalke S Irmler K Pivotal role of AtSUVH2 in heterochromatin histone methylation and gene silencing in Arabidopsis EMBO J 2005 9 1418 1429 10.1038/sj.emboj.7600604 15775980 PMC1142535

[B12] Veiseth S Rahman M Yap K Fischer A Jacobsen W Reuter G The SUVR4 histone lysine methyltransferase binds ubiquitin and converts H3K9me1 to H3K9me3 on transposon chromatin in Arabidopsis Plos Genet 2011 9 e1001325 10.1371/journal.pgen.1001325 21423664 PMC3053343

[B13] Zhang X Clarenz O Cokus S Bernatavichute YV Pellegrini M Goodrich J Whole-genome analysis of histone H3 lysine 27 trimethylation in *Arabidopsis* Plos Biol 2007 9 1026 1035 10.1371/journal.pbio.0050129 PMC1852588 17439305

[B14] Zheng B Chen X Dynamics of histone H3 lysine 27 trimethylation in plant development Curr Opi Plant Biol 2011 9 123 129 10.1016/j.pbi.2011.01.001 PMC3081887 21330185

[B15] Thorstensen T Grini PE Aelen RB SET domain proteins in plant development Biochim Biophys Acta 1809 9 407 420 10.1016/j.bbagrm.2011.05.008 21664308

[B16] Saleh A Alvarez R Avramova Z An efficient chromatin immunoprecipitation (Chip) protocol for studying histone modifications in *Arabidopsis* plants Nat Prot 2010 9 1018 1025 10.1038/nprot.2008.66 18536649

[B17] Deal RB Henikoff J Henikoff S Genome-wide kinetics of nucleosome turnover determined by metabolic labeling of histones Science 2010 9 1161 1164 10.1126/science.1186777 20508129 PMC2879085

[B18] Perrella G Consiglio M Aiese R Cremona G Sanchez M Barra L Histone hyperacetylation affects meiotic recombination and chromosome segregation in *Arabidopsis* Plant J 2010 9 796 806 10.1111/j.1365-313X.2010.04191.x 20230492

[B19] Ay N Irmler K Fischer A Uhlemann R Reuter G Humbeck K Epigenetic programming via histone methylation at WRKY53 controls leaf senescence in *Arabidopsis thaliana* Plant J 2009 9 333 346 10.1111/j.0960-7412.2009.03782.x 19143996

[B20] Santamaría E Hasbún R Valera J Meijón M Valledor L Rodríguez J Acetylated H4 histone and genomic DNA methylation patterns during bud set and bud burst in *Castanea sativa* J Plant Physiol 2009 9 1360 1369 10.1016/j.jplph.2009.02.014 19376609

[B21] Meijón M Feito I Valledor L Rodríguez R Cañal MJ Dynamics of the DNA methylation and Histone H4 acetylation during floral bud differentiation in azalea BMC Plant Biol 2010 9 10 10.1186/1471-2229-10-10 20067625 PMC2923518

[B22] Smertenko A Hussey P Suárez MF, Bozhkov PV Immunolocalization of proteins in somatic embryos Plant Embryogenesis 2008 Totowa: Humana Press 157 171

[B23] Yang X Yuan L Makaroff C Pawloski W Immunolocalization protocols for visualizing meiotic proteins in Arabidopsis thaliana: Method 3 Plant Meiosis, Methods in Molecular Biology 2013 New York: Springer+Business Media 109 118 10.1007/978-1-62703-333-6_11 23559207

[B24] Shi S Key M Kalra K Antigen retrieval in formalin-fixed, paraffin-embedded tissues: an enhancement method for immunohistochemical staining based on microwave oven heating of tissue sections J Histochem Cytochem 1991 9 741 748 10.1177/39.6.1709656 1709656

[B25] Jasencakova Z Soppe W Meister A Turner B Schubert I Histone modifications in Arabidopsis-high methylation of H3 lysine 9 is dispensable for constitutive heterochromatin Plant J 2003 9 471 478 10.1046/j.1365-313X.2003.01638.x 12581305

[B26] Hou Z Huang W Immunohistochemical localization of IAA and ABP1 in strawberry shoot apexes during floral induction Planta 2005 9 678 687 10.1007/s00425-005-0014-1 16001261

[B27] Yao X Feng H Yu Y Dong A Shi Y Shen WH SDG2-Mediated H3K4 methylation is required for proper *Arabidopsis* root growth and development Plos One 2013 9 e56537 10.1371/journal.pone.0056537 23483879 PMC3585709

[B28] Friml J Benkova E Mayer U Palme K Muster G Automated whole mount localization techniques for plant seedlings Plant J 2003 9 115 124 10.1046/j.1365-313X.2003.01705.x 12662314

[B29] Shi S Cote R Taylor C Antigen retrieval techniques: current perspectives J Histochem Cytochem 2001 9 931 937 10.1177/002215540104900801 11457921

[B30] Koltai H Bird D High throughput cellular localization of specific plant mRNAs by liquid-phase in situ reverse transcription-polymerase chain reaction of tissue sections Plant Physiol 2000 9 1203 1212 10.1104/pp.123.4.1203 10938339 PMC1539267

[B31] Shi S Cheng Q Zhang P Wang N Zheng Y Bai X Immunoflourescence with dual microwave of paraffin-embedded sections in the assessment of human renal biopsy specimens Am J Clin Pathol 2013 9 71 78 10.1309/AJCPRZG8EXN7BAID 23270901

[B32] Shi S Zhang P Cheng Q Wu J Cui J Zheng Y Immunohistochemistry of deparaffinized sections using antigen retrieval with microwave combined pressure cooking versus immunoflourescence in the assessment of human renal biopsies J Clin Pathol 2013 9 374 380 10.1136/jclinpath-2012-201125 23476077

[B33] Benhamou N Noel S Grenier J Asselin A Microwave energy fixation of plant tissue: an alternative approach that provides excellent preservation of ultrastructure and antigenicity J Elec Microsc Tech 1991 9 81 94 10.1002/jemt.1060170109 1993940

[B34] Medina FJ Cerdido A Maroto M Manzanares M Marco R Enhancement of the immunocytochemical detection of antigens by microwave irradiation. Benefits and limitations analyzed in isolated plant nuclei and Drosophila embryos in toto Histochemistry 1994 9 45 50 10.1007/BF00271048 7814269

[B35] Lería F Marco R Medina FJ Structural and antigenic preservation of plant samples by microwave-enhanced fixation, using dedicated hardware, minimizing heat-related effects Microsc Res Tech 2004 9 86 100 10.1002/jemt.20109 15570593

[B36] Sibony M Commo F Callard P Gasc J Enhancement of mRNA in situ hybridization signal by microwave heating Lab Invest 1995 9 586 591 7474931

[B37] Nic-Can G De la Peña C Loyola-Vargas VM, Ochoa-Alejo N Determination of histone methylation in mono- and dicotyledonous plants Plant Cell Culture Protocols, Methods in Molecular Biology 2012 Heidelberg: Humana Press 313 324 10.1007/978-1-61779-818-4_24 22610638

[B38] De-la-Peña C Nic-Can G Ojeda G Herrera J Lopez Torres A Wrobel K *KNOX1* is expressed and epigenetically regulated during *in vitro* conditions in *Agave spp* BMC Plant Biol 2012 9 203 10.1186/1471-2229-12-203 23126409 PMC3541254

[B39] Jackson JP Johnson L Jasencakova Z Zhang X Burgos L Singh P Dimethylation of histone H3 lysine 9 is a critical mark for DNA methylation and gene silencing in *Arabidopsis thaliana* Chromosoma 2004 9 308 315 10.1007/s00412-004-0275-7 15014946

[B40] Borg M Brownfield L Twell D Male gametophyte development: a molecular perspective J Exp Bot 2009 9 1465 1478 10.1093/jxb/ern355 19213812

[B41] Murashige T Skoog F A revised medium for rapid growth and bioassays with tobacco tissue cultures Physiol Plant 1962 9 473 497 10.1111/j.1399-3054.1962.tb08052.x

[B42] Mordhorst A Hartog M El Tamer M Laux T De Vries S Somatic embryogenesis from *Arabidopsis* shoot apical meristem mutants Planta 2002 9 829 836 10.1007/s00425-001-0700-6 11941458

[B43] Quiroz-Figueroa FR Monforte-González M Galaz-Avalos RM Loyola-Vargas VM Loyola-Vargas VM, Vázquez-Flota FA Direct somatic embryogenesis in *Coffea canephora* Plant cell culture protocols 2006 Totowa, New Jersey: Humana Press 111 117

