# Supplementary figures and images for: An efficient immunodetection method for histone modifications in plants
Source: Plant Methods. 2013 Dec 16;9:47. doi: 10.1186/1746-4811-9-47 (PMC3868413; doi:10.1186/1746-4811-9-47)

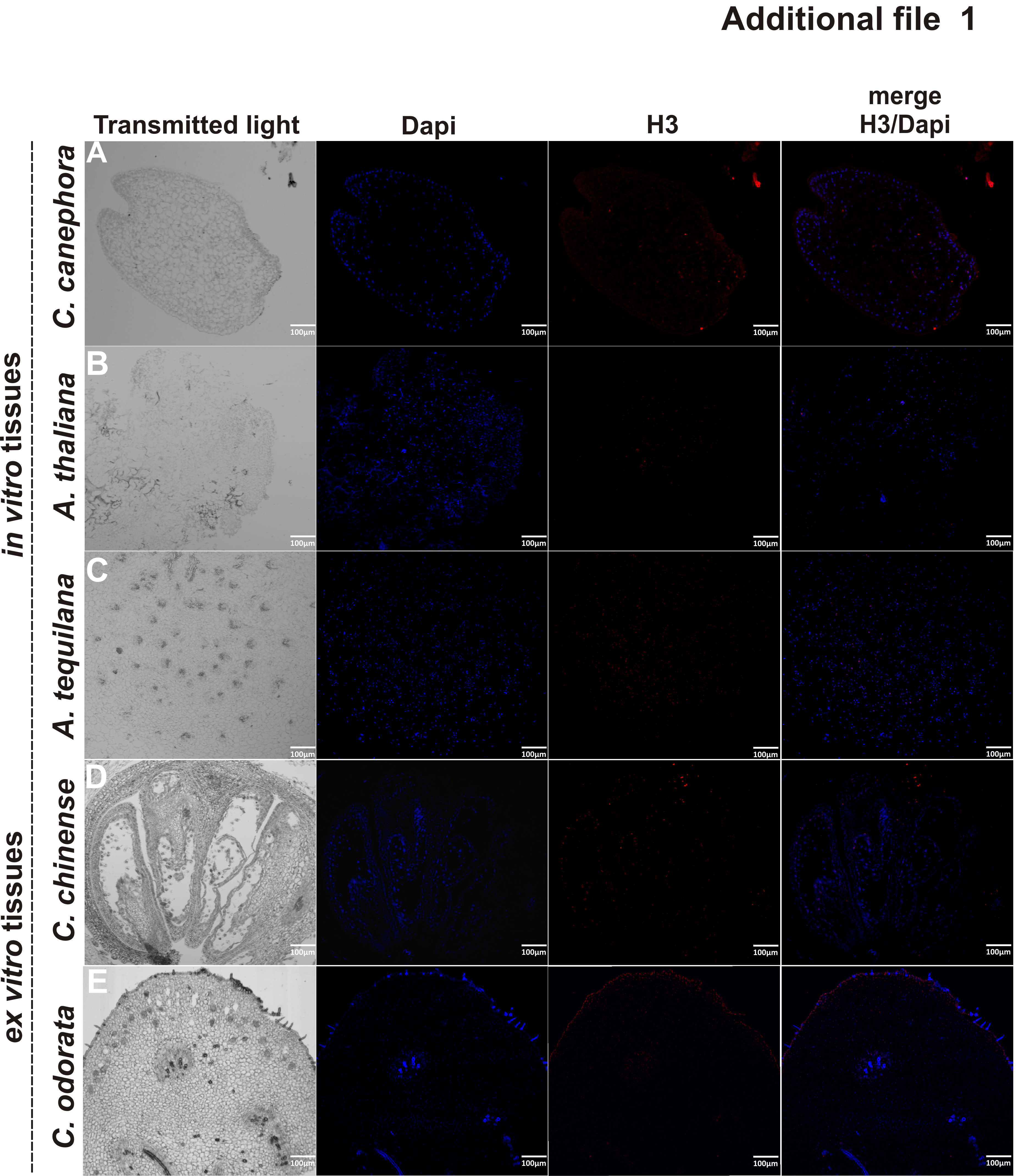

Supplement: Additional file 1 — Immunodetection of histone H3 in different plant species tissues without the microwave treatment (negative control). Immunodetection against histone H3 avoiding the antigen retrieval from the microwaved citrate buffer in the globular embryo of Coffea canephora (A), meristematic zones in the callus of Arabidopsis thaliana (B), procambium zone of Agave tequilana (C), anthers of Capsicum chinense (D) and bud of Cedrela odorata (E). [file 1746-4811-9-47-S1.tiff]

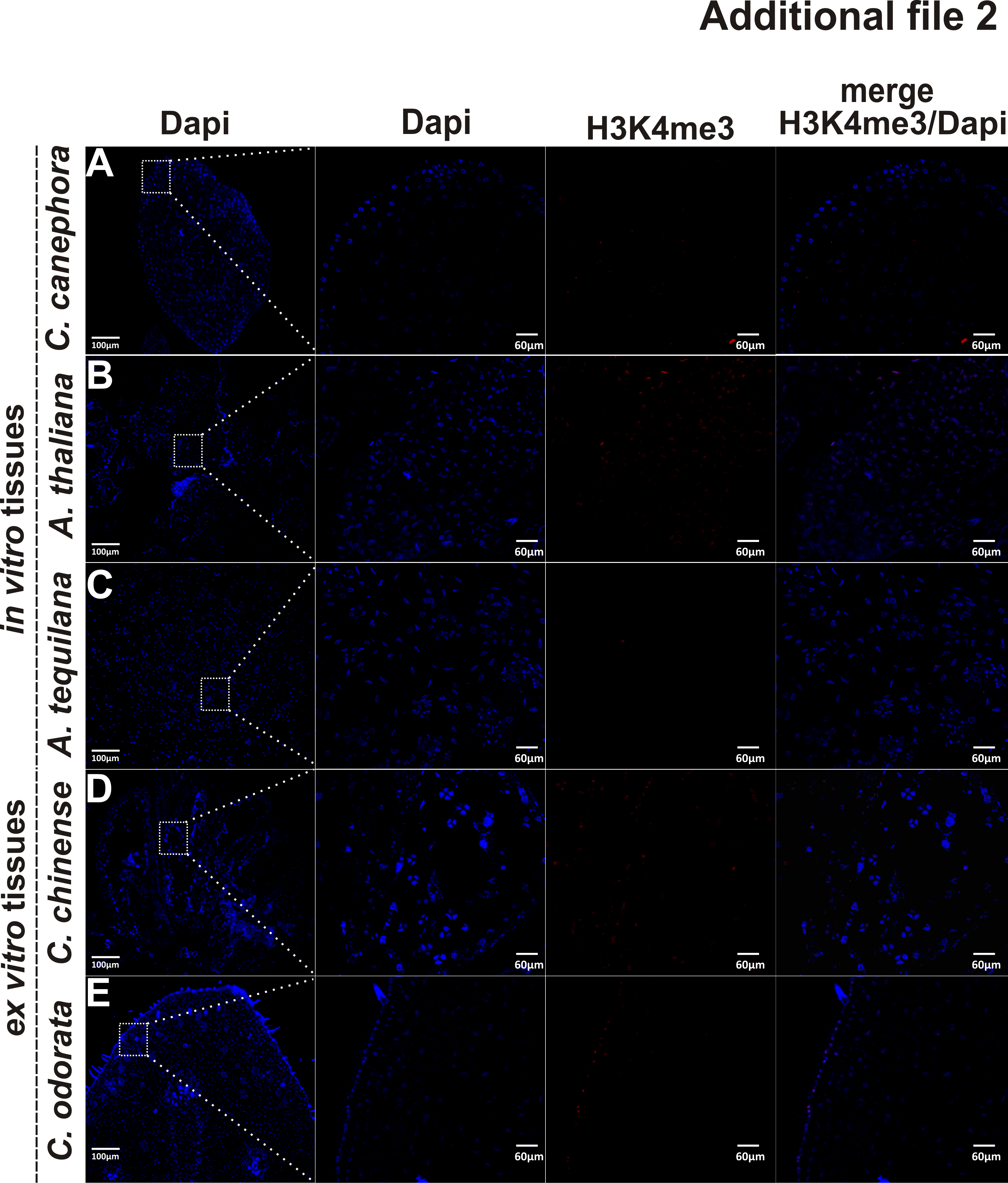

Supplement: Additional file 2 — Immunodetection of H3K4me3 in different plant species tissues without the microwave treatment (negative control). Immunodetection against H3K4me3 avoiding the antigen retrieval from the microwaved citrate buffer in the epidermis cells of globular embryo of Coffea canephora (A), meristematic zones in the callus of Arabidopsis thaliana (B), shoot apex of Agave tequilana (C), anthers of Capsicum chinense (D) and in the proximal cells to epidermis of Cedrela odorata (E). Dashed squares represent the close-up of the sites analyzed by immunodetection against H3K4me3 without antigen retrieval. [file 1746-4811-9-47-S2.tiff]

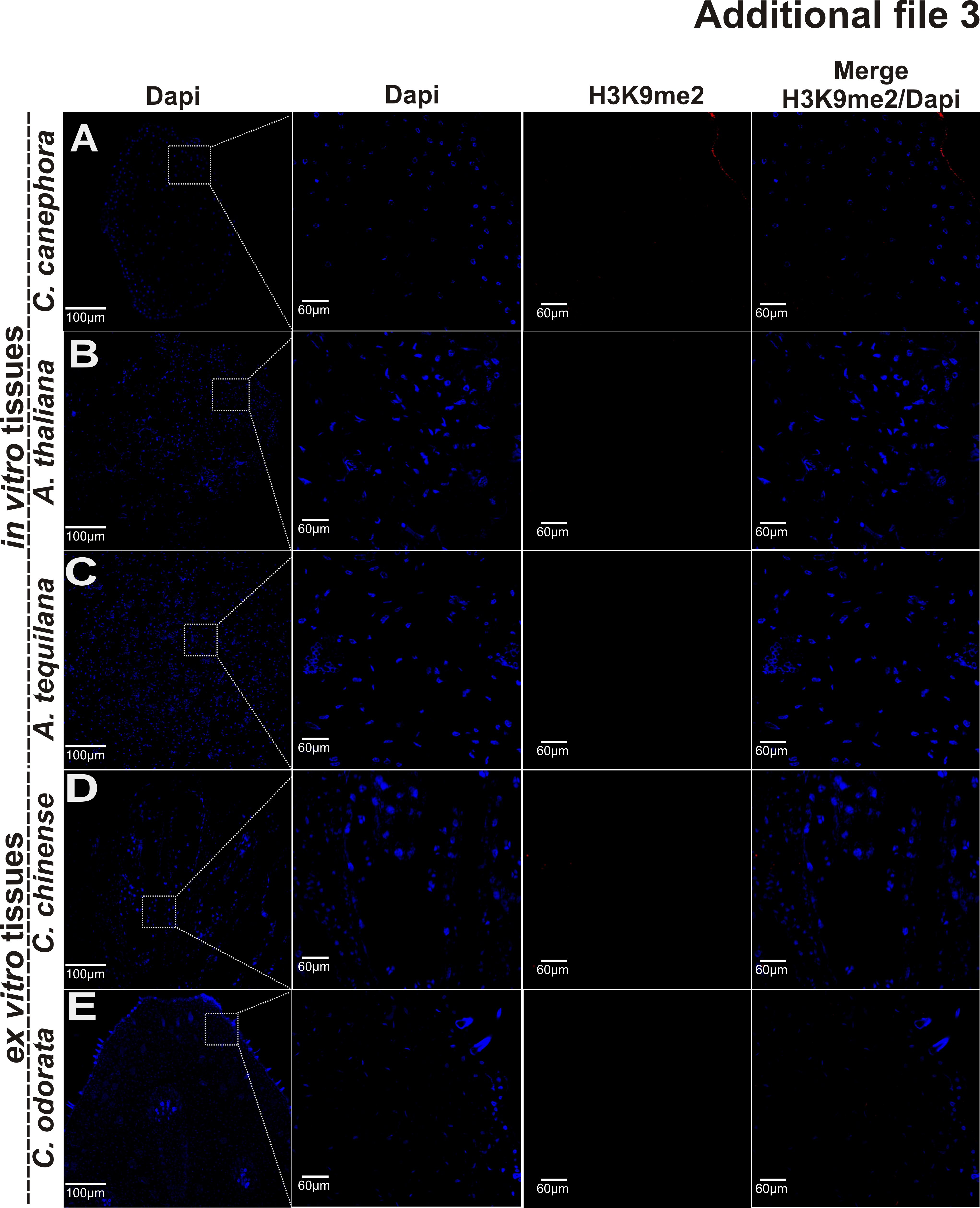

Supplement: Additional file 3 — Immunodetection of H3K9me2 in different plant species tissues without the microwave treatment (negative control). Immunodetection against H3K9me2 avoiding the antigen retrieval from the microwaved citrate buffer in the epidermis of globular embryo of Coffea canephora (A), meristematic zones in the callus of Arabidopsis thaliana (B), shoot apex of Agave tequilana (C), anthers of Capsicum chinense (D) and in the proximal cells to epidermis of Cedrela odorata (E). Dashed squares represent the close-up of the sites analyzed. [file 1746-4811-9-47-S3.tiff]
